# Can negative HRCT locate ethmoid CSF leaks? A retrospective analysis

**DOI:** 10.3389/falgy.2026.1914144

**Published:** 2026-08-28

**Authors:** Jie-Wen Zhou, Xin-Yu Zhang, Yu-Wen Gong, Jing-Wen Yuan, Meng-Qi Zhao, Xiao-Bai Pang, Jian Li, Wei-Ping Wen, Nian-Zhen Zheng

**Affiliations:** 1 Department of Otorhinolaryngology, the First Affiliated Hospital of Sun Yat-sen University, Guangzhou, China; 2 Otorhinolaryngology Institute, Sun Yat-sen University, Guangzhou, Guangdong, China; 3 Guangzhou Key Laboratory of Otorhinolaryngology, Guangzhou, Guangdong, China; 4 Guangxi Hospital Division, the First Affiliated Hospital, Sun Yat-sen University, Nanning, China; 5 Department of Allergy, First Affiliated Hospital of Sun Yat-sen University, Guangzhou, Guangdong, China; 6 Guangxi Academy of Medical Sciences and the People’s Hospital of Guangxi Zhuang Autonomous Region, Nanning, Guangxi, China; 7 Department of Otorhinolaryngology, the Seventh Affiliated Hospital of Sun Yat-sen University, Shenzhen, China

**Keywords:** endoscopic exploration, ethmoid region cerebrospinal fluid rhinorrhea, imaging examination, skull base defect localization, spontaneous cerebrospinal fluid rhinorrhea

## Abstract

**Objective:**

Spontaneous cerebrospinal fluid (CSF) rhinorrhea is a rare condition, and preoperative identification of the defect location can be challenging. This study aims to summarize the clinical characteristics of spontaneous CSF rhinorrhea and analyze the relationship between ethmoid region defects and missed detection on high-resolution computed tomography (HRCT).

**Data sources:**

We included patients diagnosed with spontaneous CSF rhinorrhea via endoscopic surgery from 2016 to 2024 at the First Affiliated Hospital, Sun Yat-sen University.

**Review methods:**

Retrospective cohort study. Descriptive statistical analysis was conducted to summarize clinical characteristics. The t-test and Fisher's exact test were used for comparison between patients with defects located in the ethmoid region and those without. Multifactorial logistic regression analysis was performed to evaluate the relationship between ethmoid region defects and HRCT missed detection.

**Results:**

A total of 49 patients were included in the study, comprising 20 males (40.82%) and 29 females (59.18%). The average BMI was 25.68 kg/m^2^. Common symptoms included clear rhinorrhea (89.80%) and headache (20.41%). Multifactorial logistic regression revealed that patients with ethmoid region defects had a 0.04 times lower likelihood of HRCT detection (OR 0.06, 95% CI 0.00–0.40) compared to those with defects located elsewhere.

**Conclusion:**

Ethmoid region is the most common site of spontaneous CSF rhinorrhea. Pre-operative detection of rhinorrhea defects using HRCT, the most commonly used imaging method in clinical practice, is challenging. Endoscope exploration is necessary for patients who had suspicious symptoms.

## Introduction

Spontaneous cerebrospinal fluid (CSF) leaks occur with an incidence of approximately 5 per 10,000 individuals, with nearly 40% of cases being non-traumatic (1). Among these, spontaneous CSF rhinorrhea is the predominant form and is frequently associated with idiopathic intracranial hypertension (IIH). This condition is characterized by a non-negligible recurrence rate (2, 3). As the prevalence of obesity rises, spontaneous CSF rhinorrhea has gained increasing attention within both rhinology and neurosurgery (4). β2-transferrin testing is widely used in clinical practice for qualitative diagnosis of spontaneous CSF rhinorrhea, with a reported sensitivity ranging from 87% to 100% and specificity from 71% to 100% (5).

Accurate localization of the defect is also crucial for the diagnosis and management of spontaneous CSF rhinorrhea (6, 7). The ethmoid roof and cribriform plate are the most common sites of defect, followed by the sphenoid lateral recess and sphenoid roof, and the posterior wall of the frontal sinus was the least affected (8). High-resolution CT (HRCT) is a simple and essential imaging modality for diagnosis, while endoscopy can provide preliminary assessment based on the direction of fluid drainage. However, as the most widely used imaging technique, HRCT exhibits a sensitivity range of 58.8%–100% and specificity between 57% and 100%. Despite its widespread use, HRCT has limitations, particularly in cases with multiple defects (6).

MRI is also utilized in clinical practice, with 3D MRI and heavily weighted T2W MRI significantly improving defect detection. However, the utility of these techniques is limited by the hardware capabilities of medical institutions (9). CT or MRI cisternography, while effective for defect localization, are more invasive, less sensitive, and associated with potential complications (1). Intrathecal fluorescein administration is an intraoperative method for CSF rhinorrhea defect localization. Its sensitivity increased with increasing fluorescein concentration (73.8%–92.9%). Nevertheless, meta-analysis indicated that 60% of patients who underwent this procedure would experience at least one of the measured complications (10).

Despite the availability of various diagnostic methods, some patients still present with unidentified defects before surgery, requiring surgical exploration (11). The cribriform plate and ethmoid roof, being the most fragile parts of the anterior skull base, are often excessively pneumatized and contain numerous natural channels. This anatomical complexity can make defect recognition via imaging, particularly HRCT, challenging (11). In this article, we aim to summarize the clinical characteristics of spontaneous CSF rhinorrhea and investigate whether defects in the ethmoid skull base pose a particular challenge for HRCT detection, suggesting the need for further imaging investigation and possibly surgical exploration.

## Methods

### Patient inclusion

This retrospective study included patients diagnosed with spontaneous CSF rhinorrhea who were admitted to the otolaryngology department of the First Affiliated Hospital, Sun Yat-sen University, for CSF rhinorrhea repair surgery from January 1, 2016, to December 31, 2024. The inclusion criteria for spontaneous CSF rhinorrhea were:

1. Clear rhinorrhea with a glucose concentration greater than 1.7 mmol/L.
2. HRCT indicating a suspected skull base bone defect.
3. Nasal endoscopy showing clear rhinorrhea from the middle meatus or olfactory cleft.
4. In the absence of the above features, a medical history of clear rhinorrhea (differentiated from allergic rhinitis), meningitis, or aspiration pneumonia was required for empirical diagnosis.

Surgical confirmation was necessary to validate the diagnosis. Patients with a confirmed etiology such as trauma or tumors were excluded from the study.

### Clinical and imaging data collection and subgroup analysis

Demographic data, clinical symptoms, signs, and endoscopic findings were collected from the medical record system. Two radiologists with over 10 years of experience reviewed the preoperative CT and MR images of these patients. The radiologists confirmed the CSF rhinorrhea-related imaging findings and the location of the skull base defects. The CSF rhinorrhea repair surgeries were performed by experienced rhinologists (12), and the final defect localization was confirmed through surgical records.

To assess the diagnostic performance of HRCT, MRI, and their combination for detecting ethmoid region CSF rhinorrhea, a subgroup comparative analysis was conducted. Patients with defects located at the ethmoid roof and cribriform plate were classified as having ethmoid region CSF rhinorrhea. Patients with defects in the posterior wall of the frontal sinus, sphenoid roof, or sphenoid lateral recess were classified as not having ethmoid region CSF rhinorrhea.

### Statistical analysis

Statistical analysis was conducted using R version 4.5.1 (2025-06-13 UCRT). For the description of clinical characteristics and subgroup comparisons, continuous variables were expressed as means (SD), and t-tests were used for differential analysis. Categorical variables were presented as frequencies, with Fisher's exact test used for comparison. Logistic regression was applied to assess risk factors for categorical dependent variables.

## Results

According to the inclusion criteria, a total of 49 patients were included in our study, consisting of 20 males (40.82%) and 29 females (59.18%). The average BMI was 25.68 kg/m^2^, which qualified as overweight. Most of the patients (91.84%) had a history of CSF rhinorrhea-related symptoms lasting more than one month. The most common symptoms were clear rhinorrhea (89.80%) and headache (20.41%), while symptoms such as nasal congestion, anosmia, and central nervous system infections were relatively rare.

To evaluate the efficacy of different diagnostic methods, we calculated the detection rates of CSF rhinorrhea using endoscopy, CT, and MRI. The detection rates were 63.41% (CT), 68.29% (MRI), and 77.14% (endoscopy), respectively. However, all methods had limitations in CSF rhinorrhea detection and defect localization. Image examination revealed 19 patients (38.78%) with meningoencephalocele and 8 patients with empty sella (Table 1).

**Table 1:** Clinical characteristics of spontaneous CSF rhinorrhea patients.

		Group		*P* value
Clinical characteristics	Total (*n* = 49)	Location in ethmoid (*n* = 32)	Location not in ethmoid (*n* = 17)	
Gender				0.9632
Male	20 (40.82%)	12 (37.50%)	8 (47.06%)	
Female	29 (59.18%)	20 (62.50%)	9 (52.94%)	
Age	40.36 ± 18.38 (yrs)	39.65 ± 19.34 (yrs)	41.71 ± 16.91 (yrs)	0.7131
Height	1.55 ± 0.21 (m)	1.56 ± 0.20 (m)	1.53 ± 0.23 (m)	0.7876
Weight	63.48 ± 19.50 (kg)	63.96 ± 20.41 (kg)	64.78 ± 22.57 (kg)	0.8472
BMI	25.68 ± 4.74 (kg/m2)	25.82 ± 4.70 (kg/m2)	25.42 ± 4.97 (kg/m2)	0.8021
Duration				0.9344
<1m	4 (8.16%)	1 (3.13%)	3 (17.65%)	
1–3m	10 (20.41%)	8 (25.00%)	2 (11.76%)	
3–6m	12 (24.49%)	7 (21.88%)	5 (29.41%)	
6m−1y	10 (20.41%)	7 (21.88%)	3 (17.65%)	
>1y	13 (26.53%)	9 (28.13%)	4 (23.53%)	
Symptom				
Headache	10 (20.41%)	5 (15.63%)	5 (28.41%)	0.0908
Congestion	5 (10.20%)	4 (12.50%)	1 (5.88%)	0.0139
Anosmia	0 (0.0%)	0 (0.00%)	0 (0.00%)	
Infection	2 (4.08%)	1 (3.13%)	1 (5.88%)	0.0022
Rhinorrhea	44 (89.80%)	28 (87.50%)	16 (94.12%)	0.5360
Meningoencephalocele	19 (38.78%)	12 (37.50%)	7 (41.18%)	0.5670
Rhinorrhea side	(*n* = 44)	(*n* = 28)	(*n* = 16)	0.0425
Unilateral	43 (97.73%)	28 (100.00%)	15 (93.75%)	
Bilateral	1 (2.27%)	0 (0.00%)	1 (6.25%)	
CT rhinorrhea	26 (63.41%) (*n* = 41)	12 (48.00%) (*n* = 25)	14 (87.50%) (*n* = 16)	0.0179
Empty sella	8 (19.51%) (*n* = 41)	5 (20%) (*n* = 25)	3 (18.75%) (*n* = 16)	0.0731
MR rhinorrhea	28 (68.29%) (*n* = 41)	16 (64.00%) (*n* = 25)	12 (75.00%) (*n* = 16)	0.4624
Endoscope rhinorrhea	27 (77.14%) (*n* = 35)	18 (78.26%) (*n* = 23)	9 (75.00%) (*n* = 12)	0.8275
Defect diameter	0.75 ± 0.56 (cm) (*n* = 23)	0.67 ± 0.58 (cm) (*n* = 17)	0.94 ± 0.52 (cm) (*n* = 6)	0.2925

To analyze the correlation between defect location in the ethmoid region and HRCT findings, we divided the patients into two groups: one with defects confirmed to be located in the ethmoid region, and the other with defects outside this region. Patients with defects in the ethmoid region had lower rates of headache (5 (15.63%) vs. 5 (28.41%)) and a higher incidence of empty sella (5 (20%) vs. 3 (18.75%)). These patients were also more difficult to detect with HRCT (12 (48%) vs. 14 (87.50%)).

Further, multivariate logistic regression analysis showed that, after adjusting for confounding factors such as headache and empty sella, patients with defects located in the ethmoid region had 0.04 times the risk of being detected by HRCT (OR = 0.06, 95% CI 0.00–0.40) (Table 2).

**Table 2:** Multiple logistic regression analysis of influencing factor for CSF rhinorrhea ethmoid region location.

Variables	OR (95% CI)	*P* value	Regression coefficient
CT rhinorrhea	0.06 (0.00–0.40)	0.0146	−2.793
Headache	0.09 (0.00–0.76)	0.0535	−2.388
Empty sella	0.65 (0.09–4.45)	0.6490	−0.437

**Figure 1:**
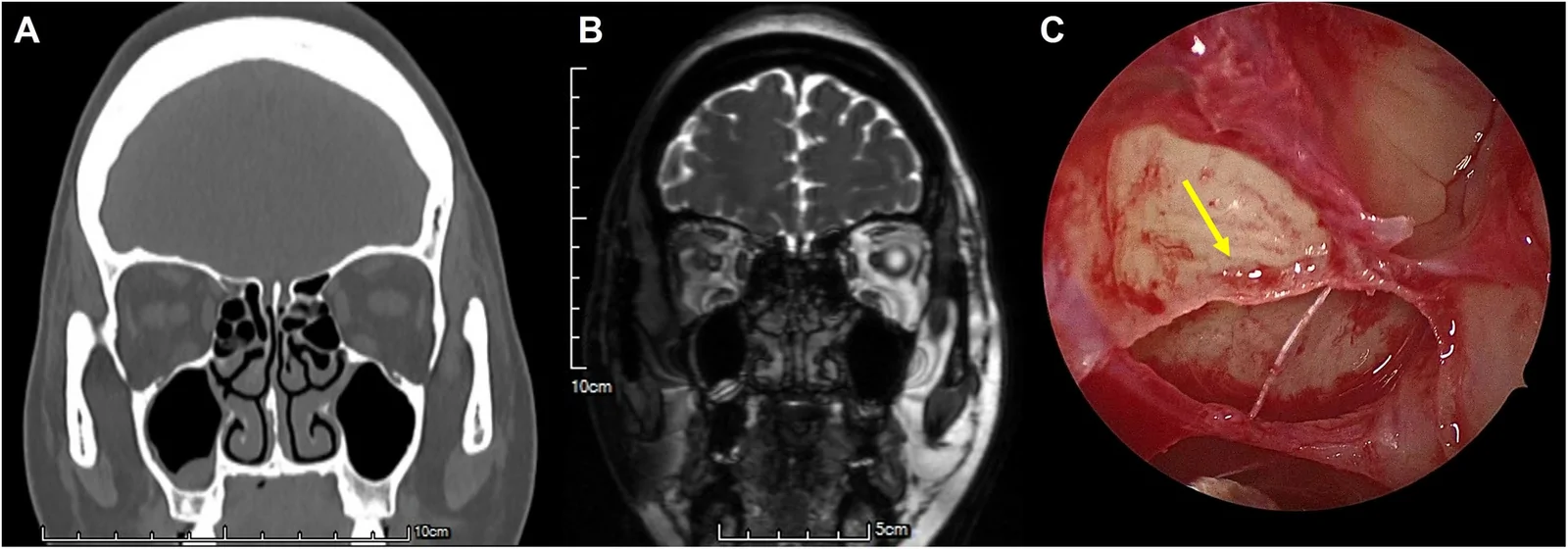
A confirmed ethmoid CSF leak not identified on HRCT **(A)** or T2W MRI **(B)** endoscopy **(C)** located the defect anterior to the anterior ethmoid artery (arrow).

A typical case is presented below. This patient was a 48-year-old woman who was admitted with a headache lasting over one month. She also reported postnasal drip with intracranial pressure of 82 mm H₂O. After excluding trauma, intracranial tumors, cerebrovascular diseases, and nasal diseases, the patient was initially diagnosed with spontaneous CSF rhinorrhea. However, both HRCT and heavily weighted T2W MRI failed to provide positive indications of CSF rhinorrhea. Endoscopic surgery was performed for diagnostic confirmation and defect repair. During ethmoid sinus contouring, a slit-like defect was found on the ethmoid roof of the anterior ethmoid. After repairing the defect with middle meatus flap, the patient experienced significant improvement in headache and postnasal drip (Figure 1).

## Discussion

In this study, we reviewed the clinical data of spontaneous CSF rhinorrhea patients at our hospital from 2016 to 2024. Consistent with existing consensus, obese women comprised the majority of these patients. The ethmoid region was found to be the most common site of CSF rhinorrhea (65.3%), as previously reported. Among the patients with ethmoid region CSF rhinorrhea, we observed a lower incidence of headache, which is a typical symptom of idiopathic intracranial hypertension (IIH), and it was more challenging to detect the skull base defect using HRCT.

The relationship between spontaneous CSF rhinorrhea and IIH had been widely discussed. IIH was characterized by intracranial hypertension without structural intracranial or CSF abnormalities, typically presenting with headache and papilledema (4, 13). In clinical data from China, 75.8% of spontaneous CSF rhinorrhea patients were found to have elevated intracranial pressure (ICP) (>180 mmHg), although only a few were diagnosed with IIH (2). Another cohort study conducted in Spain reported a mean ICP of 33.0 cm H2O (range: 20–55 cm H2O), which was also above the normal range (14). Chronic intracranial hypertension can gradually cause bone erosion, leading to CSF leaks and meningoencephalocele at weak areas, such as the cribriform plate, ethmoid roof, and sphenoid lateral recess (14). The natural channels in the cribriform plate, excessive pneumatization of the ethmoid roof, and height differences between these regions contribute to this weakness (15).

We hypothesized that, unlike leakage in the sphenoid lateral recess, which usually had a distinct fistula easily detected by HRCT, the presence of natural channels and the junction between the cribriform plate and ethmoid roof creates favorable conditions for CSF leak formation, leading to difficulties in defect identification. Our data indicated that ethmoid region leakage was 2.8 times harder to detect by HRCT, supporting our hypothesis. This finding suggests that the ethmoid region should be considered when spontaneous CSF rhinorrhea is suspected, even in the absence of clear imaging indications.

Imaging techniques have improved significantly over the past two decades. High-resolution MRI techniques, such as fluid-attenuated inversion recovery (MRI-FLAIR) and constructive interference steady state (CISS), help differentiate between CSF and inflammation/edema (1). Intrathecal CSF cisternography has proven to be an important adjunct for detecting CSF leaks. Various types of cisternography, including CT cisternography, MR cisternography, and radionuclide cisternography, have been reported with sensitivities of 37.5–73.2%, 56–95.65%, and 76%, respectively (6). Due to their varying sensitivity, invasiveness, radiation exposure, and potential complications, CSF cisternography is limited in clinical use (6, 7). For non-invasive alternatives, heavily weighted T2W and 3D gradient sequence MRI have been introduced with sensitivities ranging from 75% to 100% (16). A combined imaging approach, such as CT and MRI, can improve the detection efficacy of CSF rhinorrhea, with sensitivities ranging from 89.74% to 95%, making it a practical and cost-effective approach (6). In our study, 36 patients (79%) were pre-operatively evaluated using combined imaging methods. However, conflicting results between CT and MRI often created confusion for surgeons.

Our study has several limitations. First, ICP was not measured in most patients, so we could not confirm whether they had elevated ICP. Second, we did not distinguish between children and adults in this cohort, which may have influenced the results due to congenital factors. Lastly, the small sample size and retrospective design limit the generalizability and strength of our findings.

In conclusion, our study showed that spontaneous CSF rhinorrhea was commonly observed in overweight, middle-aged women, with symptoms of rhinorrhea and headache. The ethmoid region was the most frequent site of CSF rhinorrhea and was more difficult to detect using HRCT. Endoscopic exploration was essential for patients with suspicious symptoms, even when imaging results were inconclusive.

## Data Availability

The raw data supporting the conclusions of this article will be made available by the authors, without undue reservation.
